# Quality improvement report: Investigating barriers in HIV testing oncology patients to optimize HIV testing practice

**DOI:** 10.1111/hiv.70140

**Published:** 2025-11-02

**Authors:** Katharine E. A. Darling, José Damas, Ana Leni Frei, Stefano Frega, May‐Lucie Meyer, Solange Peters, Matthias Cavassini, Tu Nguyen‐Ngoc

**Affiliations:** ^1^ Infectious Diseases Service, Department of Medicine Lausanne University Hospital and University of Lausanne Lausanne Switzerland; ^2^ Institute of Tissue Medicine and Pathology, University of Bern Bern Switzerland; ^3^ Medical Oncology 2, Veneto Institute of Oncology IOV‐IRCCS Padova Italy; ^4^ Medical Oncology Service, Department of Oncology Lausanne University Hospital Lausanne Switzerland; ^5^ Oncology Service, Morges Hospital Morges Switzerland

**Keywords:** HIV testing, HIV testing barriers, HIV‐associated malignancies, quality improvement

## Abstract

**Background:**

In 2010, we observed missed opportunities for earlier human immunodeficiency virus (HIV) diagnosis among people newly diagnosed with HIV attending our service. We reached out to clinical services with low HIV testing rates.

**Local Problem:**

In the oncology service, <5% of all patients seen were tested for HIV between 2010 and 2012. With the rationale of excluding HIV‐related immunosuppression prior to prescribing immunosuppressive treatment, we aimed to identify barriers to HIV testing (Plan).

**Methods:**

In 2013, we conducted the Investigating Barriers in HIV‐Testing Oncology Patients (IBITOP I) study among people newly diagnosed with non‐AIDS‐defining cancers (non‐ADCs) (Do). We observed that 18% of patients were offered HIV testing, 16% of physicians gave reasons for not offering testing and 91% of patients accepted testing offered (Study). The Swiss HIV testing recommendations were updated in November 2013, listing aggressive immunosuppressant treatment as a testing indication. In 2015, we organized interactive training sessions on HIV testing with oncology staff (Act) and conducted a follow‐up study, IBITOP II, to examine residual barriers to testing. The primary endpoints of IBITOP II were (1) physician HIV testing offer rates, (2) physician reasons for not offering testing and (3) patient acceptance of testing offered.

**Interventions:**

Training sessions were designed following engagement with senior oncology colleagues and covered the 2013 national testing recommendations, the rationale for excluding HIV prior to prescribing immunosuppressive treatment, the excellent prognosis of HIV on antiretroviral therapy and the practical aspects of offering HIV testing.

**Results:**

Of 423 patients of unknown HIV status with newly diagnosed non‐ADCs, 257 (60.8%) were offered HIV testing. The most frequent physician reasons for not offering testing were forgetting (19.9%), patients tested recently (19.3%) and lack of time (11.5%). Patient acceptance of testing offered was 83.2%. No HIV test was positive. Since the IBITOP II study, cancer treatment options have shifted from chemotherapy to targeted therapies or immunotherapies. Consequently, HIV is now included in baseline oncology workups, circumventing the testing barriers of forgetting and lack of time and increasing HIV testing rates to almost 100%.

**Conclusion:**

HIV testing rates at our oncology service have improved following two IBITOP studies, updated national testing recommendations and the broader oncology workup required by new therapies. By including HIV testing in the baseline workup, residual barriers to HIV testing have been circumnavigated. Modelling improvement in testing practice has stemmed from engagement with oncology colleagues, despite the fact that HIV testing is mentioned in a minority of specialist oncology guidelines.

## INTRODUCTION

Human immunodeficiency virus (HIV) infection, when diagnosed and treated with antiretroviral therapy (ART) to suppress viral replication, has become a chronic disease with a near‐normal life expectancy [1]. As people living with HIV on ART continue to age, the spectrum of co‐morbidities has changed. Among people living with HIV in high‐income settings, cancer has become the leading cause of mortality [2, 3], and the spectrum of virus‐related and ‐unrelated malignancies has changed [4]. Cancers previously described as AIDS‐defining have become less frequent while the incidence of non‐AIDS‐defining cancers (non‐ADCs), including a shift towards age‐related cancers such as prostate, breast and colorectal, has increased [5].

While there is debate about the best methods of screening for cancer in people living with HIV, there are few studies on screening for HIV in people living with cancer [6, 7]. Not proposing HIV testing in people with cancer represents a potential missed opportunity for HIV diagnosis and, in people with undiagnosed HIV, can lead to suboptimal cancer management [8]. The D:A:D/RESPOND collaboration report that individuals with suboptimal immune recovery following late HIV diagnosis experience an increased risk of incident cancers, despite achieving durable virological suppression [9], highlights the importance of early HIV diagnosis.

**FIGURE 1:**
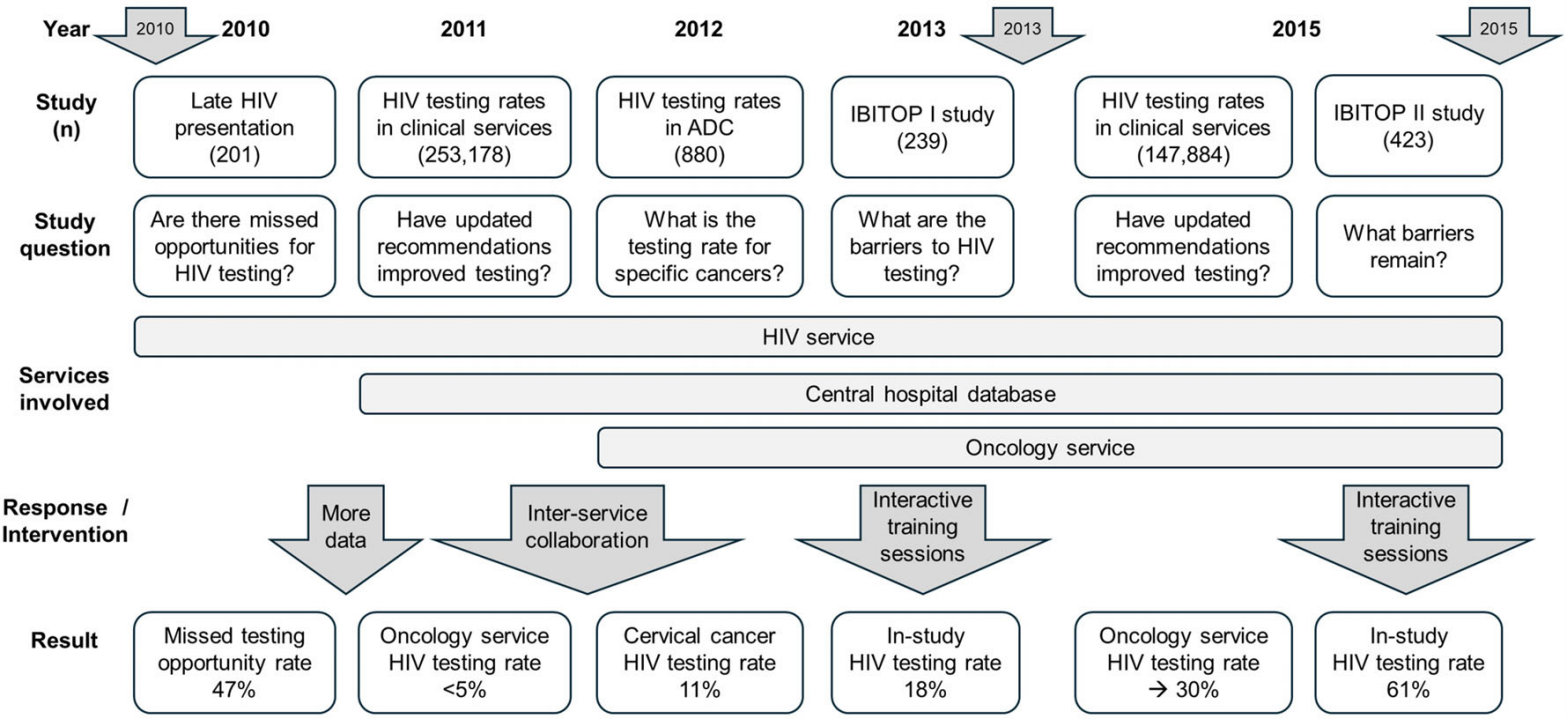
Overview of the quality improvement process arising from collaboration between the human immunodeficiency virus (HIV) and Oncology services at Lausanne University Hospital, Switzerland. The arrows (2010, 2013 and 2015) refer to the publication of national HIV testing recommendations. For each study, the (*n*) value refers to the number of participants. According to the Model of Improvement framework as reviewed by Schwartz and Rehder [10], the Study question is the improvement focus, the Response/Intervention is the idea for changing the current process and the Result is the improvement determinant. ADC, AIDS‐defining cancer.

In this quality improvement report, we describe the process of developing and implementing a quality improvement project to optimize HIV testing in the oncology service at Lausanne University Hospital, Switzerland, using a Model for Improvement framework and highlighting Plan‐Do‐Study‐Act (PDSA) cycles [10]. We use the Standards for Quality Improvement Reporting Excellence (SQUIRE) 2.0 structure to present this process [11]. PDSA cycles are summarized in Figure 1.

### Note on language used

The term *AIDS‐defining cancer* (ADC) was used during initial PDSA cycles as these were conducted prior to the consensus to retire this term [5]. In presenting a later cycle (the second study Investigating Barriers in HIV‐Testing Oncology Patients (IBITOP II) under Interventions: IBITOP II section below), we have maintained the term *non‐ADC* in the interest of uniformity. Also for uniformity, the term *testing* is used throughout, even if the exclusion of HIV prior to immunosuppressant treatment in a person with a non‐indicator condition cancer and a low pretest probability of having HIV could be classed as *screening*.

### Background: HIV testing practices and the effect of testing recommendations

In 2010, following a Swiss HIV Cohort Study report that 31% of people newly diagnosed with HIV had CD4 cell counts below 200 cells/mL [12], we started to examine missed opportunities for earlier diagnosis among our own patient population. We subsequently observed that, among 201 people newly diagnosed with HIV, 47% had presented at least one missed opportunity for earlier diagnosis [13]. This prompted an analysis of HIV testing practices at our hospital using two existing databases across 10 clinical services. HIV testing rates were calculated for each service as the number of tests performed, extracted from the immunology service database, per number of patients seen, extracted from the central hospital database [14]. Testing practices were studied over the period 2008–2012:2 years preceding and 2 years following the 2010 Swiss HIV testing recommendations proposing provider‐initiated counselling and testing [15]. The analysis demonstrated no change in testing practice following the publication of the HIV testing recommendations and identified services with low HIV testing rates [14].

### Local problem: Low HIV testing rates in oncology patients

During the HIV testing practice analysis described above, the oncology service had testing rates below 5% [14]. In collaboration with our oncology colleagues, we examined HIV testing among people diagnosed with cancers defined at the time as AIDS‐defining (Kaposi's sarcoma, non‐Hodgkin lymphoma and cervical cancer), for which HIV testing was indicated according to the 2010 national testing recommendations [15]. We observed testing rates of 60% in lymphoma patients (Hodgkin and non‐Hodgkin) and 11% in cervical cancer patients [7]. We hypothesized that, as ‘provider‐initiated’ HIV testing was the national recommendation, the way to optimize testing in the oncology service was to investigate provider‐related barriers to proposing HIV testing.

## METHODS

### Investigating barriers to HIV testing

**FIGURE 2:**
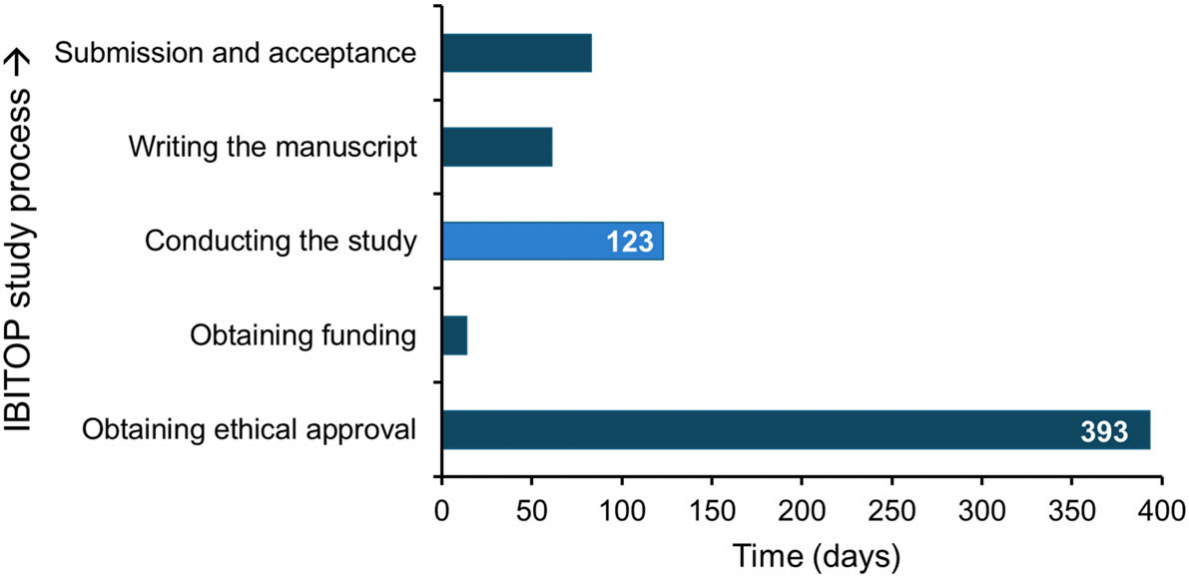
The process of conducting the first Investigating Barriers in HIV‐Testing Oncology Patients (IBITOP) study. The timeline up the *y*‐axis extends from 2012 to 2015. HIV, human immunodeficiency virus.

In 2012, we engaged with senior oncology colleagues to create a protocol for the first study IBITOP I. The main obstacle to beginning this study was obtaining permission from the cantonal ethics committee (Figure 2). Despite the rationale for excluding HIV prior to prescribing immunosuppressive treatment, it was considered too much of an additional burden for people already diagnosed with cancer to then be offered testing for HIV, another devastating diagnosis. We mention this, not as a criticism of the ethics committee, which exists after all to protect patients, but as an indication of the complex obstacles to HIV testing that existed at the time.

Permission was obtained after 393 days (Figure 2). Pre‐study interactive training sessions were designed in collaboration with oncology colleagues as we observed that only 18% of doctors in emergency departments (another service with low HIV testing rates) in French‐speaking Switzerland were aware of national HIV testing recommendations [16]. The training sessions were attended by oncology physicians and nurses and covered the 2010 HIV testing recommendations, treatment options for HIV and the practical aspects of HIV testing.

The IBITOP I study was conducted between 1st July and 31st October 2013. In this study, HIV testing was offered to 18% of 239 oncology patients newly diagnosed with non‐ADCs [17]. Although the patient testing acceptance rate was 91%, only 16% of physicians gave reasons for not testing (Table 1). Thus, while testing rates had improved, the main barriers for not testing remained unidentified.

**TABLE 1:** Physician reasons for not offering testing and patient reasons for not accepting testing in the Investigating Barriers in HIV‐Testing Oncology Patients studies (IBITOP I and II).

Physician reasons for not offering testing	IBITOP I, *n* (%)^a^	IBITOP II, *n* (%)^b^
No reason given	147 (84)	0 (0)
Forgot	1 (0.6)	33 (19.9)
Patient tested recently	‐	32 (19.3)
Second opinion (patient followed elsewhere)	10 (5.7)	28 (16.9)
No time	4 (2.3)	19 (11.5)
No chemotherapy treatment planned	‐	17 (10.2)
Excessive burden of information for patient	‐	9 (5.4)
Limited patient comprehension/language barrier	5 (2.9)	8 (4.8)
No blood test planned	3 (1.7)	6 (3.6)
Patient believed to be too old	‐	5 (3.1)
No diagnosis at time of consultation	‐	3 (1.8)
Not cancer	2 (1.1)	3 (1.8)
Patient considered not at risk	1 (0.6)	2 (1.2)
Patient refusal to any procedure	2 (1.1)	1 (0.6)

Patient reasons for not accepting testing offered	IBITOP I, *n* (%)^c^	IBITOP II, *n* (%)^d^
No reason given	‐	21 (48.8)
Patient recently tested for HIV	‐	8 (18.6)
Patient self‐perceived to be not at risk	3 (75.0)	5 (11.6)
Other	1 (25.0)	5 (11.6)
Patient followed by another physician	‐	3 (7.0)
No time	‐	1 (2.3)
Fear of being tested	‐	0 (0.0)

Abbreviation: HIV, Human immunodeficiency virus.

^a^ The number of patients not offered testing (175) is the denominator for all percentages shown.

^b^ The number of patients not offered testing (166) is the denominator for all percentages shown.

^c^ The number of patients not accepting testing (4) is the denominator for all percentages shown.

^d^ The number of patients not accepting testing (43) is the denominator for all percentages shown.

### The effect of updated HIV testing recommendations and local engagement

In November 2013, the Swiss HIV testing recommendations were updated to list aggressive immunosuppressive treatment, including chemotherapy, as an indication for HIV testing [18]. Following the publication of the 2013 recommendations, we repeated the analysis of HIV testing practices across different clinical services and observed significant increases in HIV testing rates in the services with which we had collaborated [19] (see also Figure S1). While testing rate increases were significant in the oncology service, however, it was unclear whether this was an effect of the updated national testing recommendations or of the IBITOP I study. Given the ‘justification’ for HIV testing in the 2013 recommendations, we conducted another PDSA cycle to renew engagement with oncology colleagues and identify residual barriers to HIV testing in the oncology service.

### Interventions: IBITOP II

#### Ethics statement

The IBITOP II study was approved by the Ethical Committee on Human Research of the Canton of Vaud, Switzerland (protocol number 262/11), as published previously for IBITOP I [17]. Both physicians and patients received a detailed information sheet and provided written consent prior to participating in the study. The patients' consent form included consent to HIV testing. For IBITOP II, the IBITOP I protocol was altered so that the patient's health insurance would cover the cost of HIV testing, as chemotherapy became a testing indication according to the 2013 national testing recommendations [18].

#### Setting and participants

The IBITOP II study was conducted prospectively at the oncology service at Lausanne University Hospital, Lausanne, Switzerland, between 1 January and 31 October 2015. The study employed the existing set‐up of physicians and specialist nurses, without additional staff, as described previously for IBITOP I [17].

Adult patients of unknown HIV status referred to the oncology service with a new diagnosis of solid‐organ non‐ADC, and all oncology physicians agreeing to participate were eligible for the study. Patients with ADC, recurrent neoplastic disease and those unable to provide informed consent were excluded.

#### Intervention

Prior to the study, interactive training sessions were designed with senior oncology colleagues and provided to oncology physicians and nurses by an HIV specialist (KEAD, MC). The sessions covered the 2013 Swiss HIV testing recommendations, the impact of HIV on cancer care, HIV on modern ART as a chronic medical condition, the practical aspects of offering HIV testing and the study protocol. The importance of providing reasons during the study for not offering testing, without social desirability bias or fear of judgement, was emphasized.

During the IBITOP II study, oncology physicians described the study protocol to eligible patients at their first or second clinic visit, depending on patient complexity and clinical workload. Patients were informed that participating in the study included the offer of an HIV test that would be performed on a serum sample obtained as part of the oncology workup to avoid additional blood tests and be covered by their medical insurance.

Physicians were invited to complete a short questionnaire for each patient seen, indicating whether or not they had offered HIV testing and, where applicable, to give reason(s), for not offering testing (free text, multiple reasons possible). Patient acceptance or refusal of testing offered was documented with reasons for refusal, where applicable, from a list of options (no time, not at risk, fear of result, recent test and ‘other’ with a free text option).

The oncology physician offering HIV testing would inform patients of the result, where a positive HIV test was defined as the detection of anti‐HIV‐1/2 antibodies or the HIV‐1 p24 antigen using a fourth‐generation assay (Cobas Elecsys® HIV combi PT, Roche, Rotkreuz, Switzerland). According to the study protocol, the physician would be accompanied by a specialist from the HIV outpatient clinic in the event of a positive test.

#### Measures

The primary endpoints measured were (1) oncology physician HIV testing offer rates during the initial oncology workup, (2) physician reasons for not offering testing and (3) patient acceptance of HIV testing offered.

#### Data management and statistical analysis

Patient demographic data, cancer diagnosis and questionnaire responses were entered into an anonymized database by a study investigator at the oncology service (TN‐N). Data are presented as mean ± standard deviation (SD), median ± inter‐quartile ratio (IQR) and as percentages. Statistical analysis was conducted using Stata SE 17.0 (StataCorp, College Station, TX).

## RESULTS

### Patients

During the study period, 423 people of unknown HIV status with newly diagnosed non‐ADC were seen. Median age was 64 years (54–72), 64.6% were Swiss and 70.4% were in a stable partnership. There was no significant difference in age, sex, origin (Swiss vs. non‐Swiss and by self‐identified ethnic group) or domestic setting (in couple, with children) between cancer diagnosis or between patients offered or not offered testing. Patient demographic details by cancer diagnoses are shown in Table S1.

### HIV testing offered by oncology physicians

**FIGURE 3:**
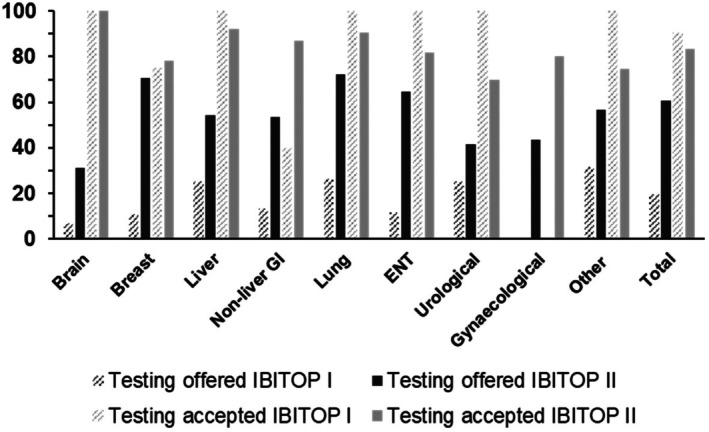
Human immunodeficiency virus (HIV) testing offer and acceptance rates by cancer diagnosis during the first and second Investigating Barriers in HIV‐Testing Oncology Patients (IBITOP) studies.

In total, 257/423 patients (60.8%) were offered HIV testing. Offer rates varied significantly by physician sex (65.8% of women offered testing compared with 49.2% of men, *p* = 0.001) but not by physician age, grade, overall medical or specialist oncological experience. The highest HIV testing offer rate was observed among patients with lung cancer (72.2%, *p* = 0.013). Physician reasons for not offering testing included forgetting, recent HIV testing, providing second opinions (patients followed elsewhere) and having no time (Table 1). HIV testing and questionnaire completion rates did not differ significantly over the study period. HIV testing offer rates by cancer diagnosis in comparison to the IBITOP I study are shown in Figure 3.

### Patient acceptance of HIV testing

Of 257 patients offered HIV testing, 214 accepted (83.2%). There were no significant demographic differences between patients accepting or declining HIV testing offered. Among the 43 patients declining HIV testing, most gave no reason (48.8%), eight (18.6%) had been tested recently and five (11.6%) did not consider themselves to be at risk (Table 1). Testing acceptance rates by cancer diagnosis in comparison to the IBITOP I study are shown in Figure 3.

### HIV prevalence

No patient tested for HIV within the IBITOP II study had a positive test.

## DISCUSSION

We have described a model of improvement in HIV testing practice occurring through identification of low testing rates and engagement with colleagues at our hospital. In the final PDSA cycle (the IBITOP II study) involving 423 patients of unknown HIV status presenting for cancer care, the rate of HIV testing offered by oncology physicians was 60.8%, up from <5% 4 years previously. The main physician reasons for not testing at this point included forgetting and time constraints. Patient acceptance of testing offered was high (83.2%). In this patient population where background HIV seroprevalence is around 0.2% [20], no test performed was positive.

The testing offer rate of 60.8% is nearly threefold higher than the rate observed in the IBITOP I study conducted prior to the inclusion of immunosuppressant treatment as a testing indication in the national testing recommendations (18%). Physician reasons for not offering testing are in line with those described elsewhere [21].

While the prevalence of undiagnosed HIV has been studied in people with cancers associated with HIV based on epidemiological studies and/or similar transmission routes (indicator conditions) [22], we could find no data on undiagnosed HIV among people with age‐associated cancers such as breast, prostate or colon, or those with lung cancer related to smoking. Equally, there are sparse studies on patient acceptance of testing. Other than IBITOP I, we identified one American study in which 80.9% of 29 549 oncology patients consented to HIV testing [23], similar to our figure of 83.2% and one study in which 92.9% of women with cervical dysplasia accepted point‐of‐care HIV testing at colposcopy outpatient clinics in the Netherlands [24].

Clinical decision support systems, whereby physicians are prompted to test when testing indications exist, and integrated testing, where HIV testing is combined with existing testing panels have been reported to improve testing when employed in hospital and nonhospital settings [25, 26]. Such systems have been made possible through large studies on indicator condition‐guided HIV testing in Europe [22, 27]. However, the difficulty of translating clinical guidance into clinical practice is well recognized [28]. Clinicians need to be aware of guidelines and agree with them before adopting and adhering (continuing to adopt) to their recommendations [28]. Many studies have described the gap between physician awareness of the indication for HIV testing and the adoption of offering testing [24], and the challenge in maintaining adoption of and adherence to guidelines over time [29, 30], while patient acceptance of HIV testing is often high [31].

Since the IBITOP II study was conducted, the cancer treatment landscape has evolved dramatically, with chemotherapy options being replaced or combined in many cases by targeted therapies or immunotherapies including immune checkpoint inhibitors [32]. Targeted therapies and immunotherapies are usually not associated with immunosuppression but the pre‐treatment workup is more complex. The European Society for Medical Oncology (ESMO) clinical practice guideline on the management of toxicities from immunotherapy lists HIV testing in the panel of tests to perform at baseline [32]. HIV testing is also mentioned in the ESMO anal cancer (‘if risk factors’) and lymphoma treatment guidelines [33, 34]. However there remains no mention of HIV testing in the European guidelines for cervical cancer [35, 36], nor for other cancers, notably lung, and head and neck cancer, outside of the use of immunotherapy in these contexts. Indeed, a recent review of non‐HIV speciality guidelines across Europe reported that fewer than half the guidelines for HIV indicator conditions recommended HIV testing [37]. Even if adherence to guidelines is imperfect, as described above, HIV testing practice will be slow to change if guidance itself for non‐HIV specialists is lacking.

In light of the changing paradigm of cancer in people living with HIV, the absence of consistent/easy‐to‐find HIV testing guidance in oncology treatment guidelines and the findings of the IBITOP II study, oncology physicians at our centre have incorporated HIV testing into the workup of cancer patients who undergo blood testing. The reason for not testing all patients at presentation is based on the experience that, during the first contact with an oncologist, cancer patients are exposed to a heavy burden of new information which may not be easy to understand or accept, such as treatment side effects or prognosis. Discussing HIV in this context, particularly when caring for patients with a poor cancer prognosis and no risk factors for HIV acquisition, is now left to the physician's clinical judgment rather than to a treatment protocol. Among patients undergoing immunosuppressive therapy, HIV testing remains mandatory and the HIV testing rate in our oncology service now approaches 100%. We believe these high testing rates result from engaging with oncology colleagues, including the PDSA cycles of the IBITOP studies, rather than an awareness of HIV testing indications. In a recent survey among oncology practitioners across France, conducted without any testing‐promoting interventions, 17% of respondents, nationally, screened for HIV routinely at the initial cancer assessment (our figure during IBITOP I) and 52% screened for HIV before immunotherapy [38]. Even if the pretest probability (risk) of a positive HIV test in our population is low, the hazard of not excluding HIV prior to planning cancer treatment is high.

This quality improvement report has limitations. Since 2015, the oncology service at our hospital has undergone restructuring, so it is no longer possible to compare HIV testing rates over time post‐2015 within a given unit. Equally, with the treatment shift towards targeted therapies and immunotherapies, some patient workups are conducted in the immunology service rather than the oncology service. It is thus not possible to review oncology service HIV testing rates using the two‐database tool described previously [14, 19] without markedly underestimating HIV testing activity. Finally, the series of PDSA cycles shown in Figure 1 has been possible through engagement and collaboration between colleagues as well as acceptance of national recommendations. Our findings therefore may not be applicable to all settings.

## CONCLUSIONS

HIV testing rates at our oncology service have improved following two IBITOP studies, updated national testing recommendations and the broader oncology workup required by new therapies. Adding immunosuppressant treatment to national HIV testing recommendations as an HIV testing indication ‘justified’ HIV testing among oncology physicians; patient acceptance of testing offered was high. By including HIV testing in the baseline oncology workup, residual barriers to HIV testing of forgetting and insufficient time have been circumnavigated. Our quality improvement experience has highlighted the importance of engaging with colleagues. High HIV testing rates among oncology patients have become possible, despite inconsistent and/or challenging‐to‐find HIV testing guidance in a minority of specialist oncology guidelines.

## AUTHOR CONTRIBUTIONS

KEAD, MC and SP conceptualized the IBITOP studies. TN‐N organized the IBITOP II questionnaire distribution and data collection. TN‐N, ALF and SF created the study database and conducted preliminary analyses for the IBITOP II study. JD collated the data and performed the final IBITOP II study analysis. M‐LM provided a review of regional oncology testing practice and M‐LM and KEAD performed a literature search. KEAD wrote the manuscript and prepared it for publication. All authors reviewed the manuscript.

## FUNDING INFORMATION

The Investigating Barriers in HIV‐Testing Oncology Patients (IBITOP) studies I and II were partially financed through research support from Gilead Sciences and an unrestricted grant from Roche. The funders had no role in the design of the study nor in the drafting of this manuscript.

## CONFLICT OF INTEREST STATEMENT

KEAD's institution has received project grants, sponsorship to attend specialist meetings and honoraria for presentations from Gilead Sciences, fees for expert opinion panels from MSD and sponsorship for educational events from Gilead Sciences, MSD and ViiV. SP has received education grants, provided consultation, attended advisory boards and/or provided lectures for the following organizations (all paid to her institution): Consultation/Advisory role: AbbVie, Amgen, Arcus, AstraZeneca, Bayer, Beigene, BioNTech, BerGenBio, Bicycle Therapeutics, Biocartis, BioInvent, Blueprint Medicines, Boehringer Ingelheim, Bristol‐Myers Squibb, Clovis, Daiichi Sankyo, Debiopharm, Eli Lilly, F‐Star, Foundation Medicine, Genmab, Genzyme, Gilead, GSK, Hutchmed, Illumina, Incyte, Ipsen, iTeos, Janssen, Qlucore, Merck Sharp and Dohme, Merck Serono, Nuvation Bio, Nuvalent, Nykode Therapeutics, Novartis, Novocure, Pharma Mar, Promontory Therapeutics, Pfizer, Regeneron, Roche/Genentech, Sanofi, Takeda and Zymeworks. Talk in a company's organized public event: AstraZeneca, Boehringer Ingelheim, Bristol‐Myers Squibb, Eli Lilly, Foundation Medicine, GSK, Illumina, Ipsen, Merck Sharp and Dohme, Novartis, Pfizer, Roche/Genentech, Sanofi and Takeda. Receipt of grants/research supports: Principal investigator in trials (institutional financial support for clinical trials) sponsored by Amgen, Arcus, AstraZeneca, Beigene, Boehringer Ingelheim, Bristol‐Myers Squibb, Eli Lilly, GSK, iTeos, Merck Sharp and Dohme, Mirati, Pharma Mar, Pfizer, Promontory Therapeutics and Roche/Genentech. MC's institution received research grants and expert opinion fees from Gilead, MSD and ViiV. MC's institution received travel grants from Gilead and ViiV. TN‐N's institution has received honoraria for providing consultation and participating in advisory boards and has received sponsorship to attend meetings for F. Hoffmann‐La Roche. The other authors have no competing interests to declare.

## Supporting information


**Figure S1.** Change in HIV testing rates in the oncology service between 2008 and 2015 measured using the two‐database tool described in the text.

**Table S1.** Characteristics of oncology patients offered HIV testing and percentage accepting HIV testing.

## Data Availability

Research data are not shared.
