# Supplementary figures and images for: Quality improvement report: Investigating barriers in HIV testing oncology patients to optimize HIV testing practice
Source: HIV Med. 2025 Nov 2;26(12):1920–9. doi: 10.1111/hiv.70140 (PMC12666251; doi:10.1111/hiv.70140)

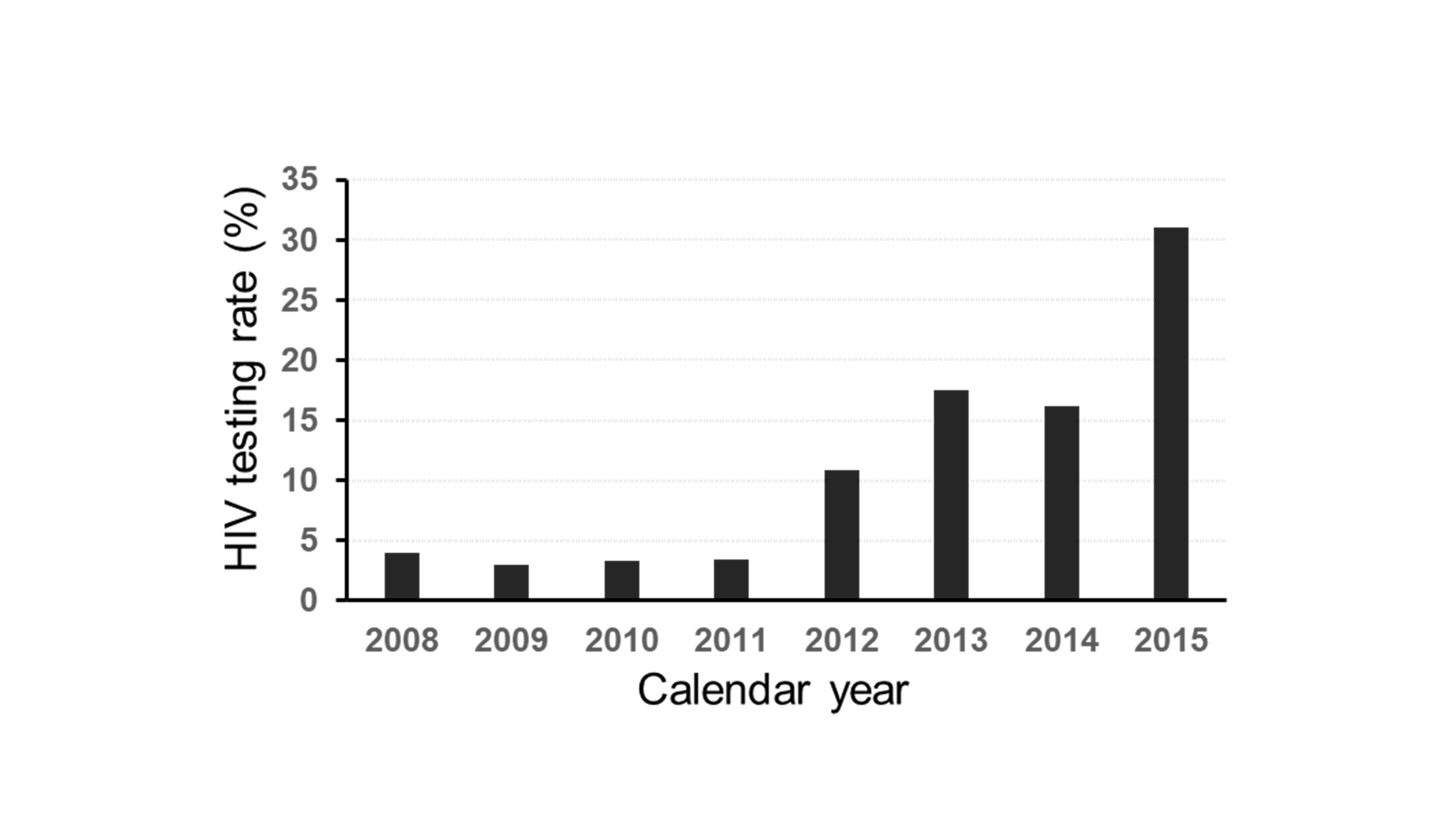

Supplement: Supplementary file 1 — Figure S1. Change in HIV testing rates in the oncology service between 2008 and 2015 measured using the two‐database tool described in the text. [file HIV-26-1920-s002.jpg]
