# Supplementary material for: Quality improvement report: Investigating barriers in HIV testing oncology patients to optimize HIV testing practice
Source: HIV Med. 2025 Nov 2;26(12):1920–9. doi: 10.1111/hiv.70140 (PMC12666251; doi:10.1111/hiv.70140)
Supplement: Supplementary file 2 — Table S1. Characteristics of oncology patients offered HIV testing and percentage accepting HIV testing. [file HIV-26-1920-s001.docx]

| Cancer type ^a^ | Brain | Breast | Liver | Non-liver gastro-intestinal | Lung | Ear, nose and throat | Urological | Gynae-cological | Other ^b^ | Total | *P-*value ^c^ |
| --- | --- | --- | --- | --- | --- | --- | --- | --- | --- | --- | --- |
| Patients offered testing of all patients seen n/N (%) | 5/16 (31.3) | 55/78 (70.5) | 13/24 (54.2) | 23/43 (53.5) | 83/115 (72.2) | 11/17 (64.7) | 10/24  (41.7) | 10/23  (43.5) | 47/83 (56.6) | 257/423 (60.8) |  |
| Patients by diagnosis,  n (%) | 5 (2.0) | 55 (21.4) | 13 (5.1) | 23 (9.0) | 83 (32.3) | 11 (4.3) | 10 (3.9) | 10 (3.9) | 47 (18.3) | 257 (100) |  |
| Age in years, median (IQR) | 45 (26;70) | 57 (48;65) | 69 (64;77) | 63 (53;71) | 65 (57;72) | 68 (60;73) | 62 (47;75) | 69 (60;75) | 64 (54;72) | 64 (54;72) | 0.55 |
| Female,  n (%) | 3 (60) | 55 (100) | 4 (30.8) | 11 (47.8) | 24 (28.9) | 3 (27.3) | 3 (30) | 10 (100) | 22 (46.8) | 135 (52.5) | 0.20 |
| Swiss,  n (%) | 3 (60) | 33 (60) | 8 (61.5) | 16 (69.6) | 50 (60.2) | 7 (63.6) | 8 (80) | 7 (70) | 34 (72.3) | 166 (64.6) | 0.81 |
| In couple,  n (%) | 1 (20) | 34 (61.8) | 10 (76.9) | 16 (69.6) | 64 (77.1) | 9 (81.8) | 8 (80) | 10 (100) | 29 (61.7) | 181 (70.4) | 0.06 |
| Patients accepting HIV testing,  n (%) | 5 (100) | 43 (78.2) | 12 (92.3) | 20 (87) | 75 (90.4) | 9 (81.8) | 7 (70) | 8 (80) | 35 (74.5) | 214 (83.2) | 0.17 |

**Table 1. Characteristics of oncology patients offered HIV testing and percentage accepting HIV testing**

^a^ Values are n (%), where the denominator is the number of patients per type of cancer, except for the row ‘Patients by diagnosis’ (where the denominator is the total number of patients offered testing, n=257, and for median age which is presented as years with interquartile range (IQR)

^b^ Other: Skin, ocular, thyroid and bone cancers

^c^ *P*-values calculated using Chi-squared or Fisher’s exact test
